# To Improve Nutrition and Healthy Eating, We Need to Generate Demand

**DOI:** 10.3389/ijph.2021.1604390

**Published:** 2021-09-21

**Authors:** Rowena K. Merritt, Puja Tshering, L. Suzanne Suggs

**Affiliations:** ^1^ University of Kent, Canterbury, United Kingdom; ^2^ Sight and Life, Basel, Switzerland; ^3^ University of Italian Switzerland, Lugano, Switzerland

**Keywords:** nutrition, demand creation, obesity, capacity building, social marketing

The world is facing a growing nutrition crisis in low, middle, and high income countries. The Global Burden of disease study (2017) found that approximately 11 million deaths were attributable to dietary risk factors, more than tobacco smoke [1].

Many low and middle-income countries struggle with a “triple burden of malnutrition” [2], where large parts of the population are either hungry, suffering from micronutrient deficiencies, and or dealing with the consequences of overweight and obesity. Globally, 149 million children are stunted (too short for their age), 50 million are wasted (too thin for their height), 340 million (or 1 in 2) suffer from deficiencies in essential vitamins and nutrients such as vitamin A and iron, and 40 million children are overweight or obese [2]. Nearly half of all deaths in children under 5 can be attributed to undernutrition [3]. Obesity is a global public health pandemic, tripling between 1975 and 2016, and causing at least 2.8 million deaths each year [4]. Excess weight drastically increases a person’s risk of developing a number of preventable NCDs, and more recently has shown to be a strong risk factor for adverse outcomes for COVID-19 [5].

Traditional approaches of informing, educating and encouraging only gets us so far. It also often blames people for their bad “habits”, preferences, or a market and system that is working against them. Thus, there needs to be a shift to a focus on supporting good nutrition by helping people make healthier choices and ensuring those healthy choices are valued and accessible. This requires coordinated, effective action by individuals, communities, organisations, industry, and policy makers to change dietary habits and social environments from “obesogenic” to supportive ones which promote healthy eating, and where people demand and prioritise nutritious foods. To generate demand for healthier foods and beverages, public health practitioners need to have a greater focus on how people’s emotions, social norms, cognitive biases, and decision-making environments all impact their behaviours and choices. A number of UN agencies and NGOs have started using Social Behaviour Change Communications (SBCC), Social Marketing, and advances in behavioural science to try and create demand for healthier diets.

Sight and Life, a Humanitarian Think Tank, has developed a number of projects and worked with partners in a “whole systems” approach to create an environment that enables healthy choices and encourages individuals to achieve and maintain a healthy diet and weight. One such initiative is OBAASIMA, developed by the project “Affordable Nutritious Foods for Women” in partnership with the German Development Cooperation (GIZ) and the private sector in Ghana. OBAASIMA aims to increase the availability of and access to new, affordable, nutritious, fortified food products for Ghanaian women. The OBAASIMA seal is placed on the packaging of affiliated products, which are then promoted through a nation-wide campaign with the slogan “Love Yourself. Stay strong for your family!”. Another project aims to increase the consumption of egg powder among pregnant and breastfeeding women, and children under 5 years of age in Ethiopia, where egg production and consumption are currently low [6]. Egg powder as an alternative is more sustainable, affordable and accessible. Working with both public and private partners in Ethiopia, the project also focuses on effective pricing and distribution strategies to create demand for egg powder, as well as establishing a sustainable market for the product.

To support more professionals in the development of effective demand-creation programmes, Sight and Life sponsored a 3-days course at the SSPH+ Summer School in Public Health 2020 and again in 2021. The course explores how tools and new innovations in behavioural science and social marketing can be used to positively impact on many behaviours, as well as to increase service uptake and generate demand for healthy goods, including healthier foods and beverages.

Capacity building and sharing of experiences across borders and between high, middle, and low income countries are paramount to tackle the triple burden of malnutrition across the globe. We hope public health organisations prioritise such training of the workforce and move from promoting healthy diets to creating a demand for healthy foods that people want, have access to, and benefit from. We call on governments, NGOs, industry, universities, and communities to prioritise programmes and policies that not only promote healthy diets, but understand the strategies that help generate demand for them. Only then, will we start to see the burden of unhealthy nutrition decrease across the world.
